# Interleukin-1 blockade in cardiac sarcoidosis: study design of the multimodality assessment of granulomas in cardiac sarcoidosis: Anakinra Randomized Trial (MAGiC-ART)

**DOI:** 10.1186/s12967-021-03130-8

**Published:** 2021-11-08

**Authors:** Jordana Kron, Thomas Crawford, Virginia Mihalick, Frank Bogun, Jennifer H. Jordan, Todd Koelling, Huzaefah Syed, Aamer Syed, Thomas Iden, Kelly Polly, Emily Federmann, Kirsta Bray, Sangeeta Lathkar-Pradhan, Shilpa Jasti, Lynda Rosenfeld, David Birnie, Melissa Smallfield, Le Kang, Alpha Berry Fowler, Amy Ladd, Kenneth Ellenbogen, Benjamin Van Tassell, W. Gregory Hundley, Antonio Abbate

**Affiliations:** 1 VCU Pauley Heart Center, Virginia Commonwealth University, Virginia Commonwealth University Medical Center, P.O. Box 980053, Richmond, VA 23298-0053 USA; 2 Cardiovascular Center, University of Michigan, Ann Arbor, MI USA; 3 Department of Biomedical Engineering, Virginia Commonwealth University, Richmond, VA USA; 4 Division of Rheumatology, Virginia Commonwealth University, Allergy, and Immunology, Richmond, VA USA; 5 Division of Pulmonary and Critical Care, Virginia Commonwealth University, Richmond, VA USA; 6 Section of Cardiovascular Medicine, Yale University School of Medicine, New Haven, CT USA; 7 University of Ottawa Heart Institute, Ottawa, ON Canada; 8 Department of Biostatistics, Virginia Commonwealth University, Richmond, VA USA; 9 Department of Pharmacotherapy and Outcomes Science, Virginia Commonwealth University, Richmond, VA USA; 10 Kenneth and Dianne Wright Center for Clinical and Translational Research, Virginia Commonwealth University, Richmond, VA USA

**Keywords:** Cardiac sarcoidosis, Interleukin-1, Inflammation, Heart failure

## Abstract

**Background:**

Sarcoidosis is an inflammatory disease characterized by the formation of granulomas, which involve the heart in up to 25% of patients. Cardiac sarcoidosis can lead to life threatening arrhythmias and heart failure. While corticosteroids have been used as a treatment for over 50 years, they are associated with hypertension, diabetes, and weight gain, further increasing cardiovascular risk. Interleukin-1 (IL-1) is the prototypical proinflammatory cytokine that works to activate the nuclear transcription factor NF-*k*B, one of the targets of glucocorticoids. IL-1 also plays an important role also in the pathophysiology of heart disease including atherosclerosis, myocardial infarction, and myocarditis.

**Methods:**

Building on a network of research collaborators developed in the Cardiac Sarcoidosis Consortium, we will investigate the feasibility and tolerability of treatment of CS with anakinra at two National Institute of Health Clinical and Translational Science Award (CTSA) hubs with expertise in cardiac sarcoidosis. In this pilot study, up to 28 patients with cardiac sarcoidosis will be recruited to compare the administration of an IL-1 blocker, anakinra, 100 mg daily on top of standard of care versus standard of care only for 28 days and followed for 180 days. Utilizing surrogate endpoints of changes in systemic inflammatory biomarkers and cardiac imaging, we aim to determine whether IL-1 blockade with anakinra can combat systemic and cardiac inflammation in patients with cardiac sarcoidosis.

**Discussion:**

The current trial demonstrates an innovative collaborative approach to clinical trial development in a rare, understudied disease that disproportionately affects females and minorities.

*Trial Registration* The trial was registered prospectively with ClinicalTrials.gov on July 12, 2019, identifier NCT04017936.

## Background

Sarcoidosis is a multisystem, granulomatous inflammatory disease seen worldwide, which disproportionately affects females and minorities, with a lifetime risk of 2.7% in black women [1, 2]. The etiology of sarcoidosis is unknown, but growing evidence suggests an inappropriate, or exaggerated, immunological response to an unidentified antigenic trigger in individuals with genetic susceptibility [3]. While more than 90% of patients have lung involvement, sarcoidosis can also affect the skin, eyes, liver, parotid gland, spleen, and other tissues and organs, including the heart. Cardiac involvement, which can occur in 25% of sarcoidosis cases, is not only less common, but also difficult to detect. Cardiac sarcoidosis can lead to life-threatening ventricular arrhythmias, heart block, heart failure, and death.

There is dearth of evidence regarding optimal treatment for cardiac sarcoidosis. There have been, indeed, no randomized clinical trials for patients with cardiac sarcoidosis. Corticosteroids are the most common therapy for sarcoidosis, yet despite more than 50 years of use, the evidence for overall efficacy from clinical trials is largely lacking, and there is no proof of survival benefit from corticosteroid treatment [4]. The use of corticosteroid treatment is also hindered by cumulative and dose-limiting toxic effects, which are largely unrelated to its anti-inflammatory effects. Corticosteroid use is associated with hypertension, diabetes, and weight gain, which can further increase cardiovascular risk, as well as osteoporosis, and increased risk of infection. There is also conflicting data on the efficacy of steroids on long-term disease outcomes [4–7]. Data suggest that routine administration of systemic steroid therapy may actually increase the possibility of relapsing disease, rather than a sustained remission [5, 6]. There is therefore an urgent need for randomized controlled trials of novel anti-inflammatory therapies in patients with cardiac sarcoidosis.

Interleukin-1 (IL-1) is the prototypical proinflammatory cytokine, also referred to as *master regulator* of the inflammatory response, involved in virtually every acute inflammatory process [8]. Pro-inflammatory stimuli such as microbes or cell debris promote the formation of the inflammasome, a macromolecular structure in the cell that functions as a scaffold for caspase-1 activation, maturation of pro-inflammatory cytokines IL-1 and IL-18, and inflammatory cell death (pyroptosis) [9, 10]. A role for IL-1 in the pathogenesis of sarcoidosis has been proposed [11]. Induction of pulmonary granuloma formation in a mouse model suggests that IL-1 participates in the pathogenesis of granuloma formation, the histologic hallmark of sarcoidosis [12]. In one study, the ratio of IL-1 receptor antagonist/IL-1β was a marker in predicting the persistence of pulmonary granulomatous lesions [11]. Patients who had improved chest imaging at 4 years had higher ratios of IL-1 receptor antagonist/IL-1β than those that did not. We previously demonstrated the presence of the NLRP3 inflammasome in the granulomas in cardiac surgical tissue of patients with cardiac sarcoidosis, providing additional support for a role of IL-1 in the pathogenesis of cardiac sarcoidosis [13]. Of note, the main mechanism of action of IL-1 is to activate the nuclear transcription factor NF-*k*B, which is also one of the therapeutic targets of glucocorticoids, the most commonly used therapy for sarcoidosis.

IL-1 also plays an important role in the pathophysiology of heart disease (i.e. acute myocardial infarction and heart failure) [14, 15] and IL-1 blockade has been evaluated as a potential therapeutic target [15]. Anakinra, recombinant human IL-1 receptor antagonist, that blocks IL-1α and IL-1β, has been FDA approved for the treatment of rheumatoid arthritis since 2001.

In 2011, the Cardiac Sarcoidosis Consortium (CSC), was founded by clinical electrophysiologists at Virginia Commonwealth University, University of Michigan, and University of Colorado with the goal to advance the understanding and treatment of cardiac sarcoidosis through collaborative clinical and translational research. The Cardiac Sarcoidosis project is an international initiative that focuses on collecting prospective demographic, clinical imaging, arrhythmias and treatment data on patients with Cardiac Sarcoidosis and also forming a network of cardiac sarcoidosis specialty hubs that may participate in future clinical trials. With 26 participating sarcoidosis centers in the U.S., Europe, and Asia, the Cardiac Sarcoidosis Consortium is a collaborative research group with over 500 patients prospectively enrolled and tracked annually within the secure web-based database.

This initial pilot study involves two Clinical and Translational Science Award (CTSA) hubs, Virginia Commonwealth University (VCU) and University of Michigan, both recognized by the World Association for Sarcoidosis and Other Granulomatous Disorders (WASOG) as WASOG Sarcoidosis Centers, with VCU being one of 25 WASOG Centers of Excellence in the world.

This pilot study addresses the feasibility of enrollment of a diverse population affected by clinically active CS, a condition that disproportionately affects women and minorities, and address the tolerability of treatment with anakinra, at two CTSA hubs and WASOG center with expertise in CS. The study aimed to explore the central hypothesis that IL-1 blockade with anakinra can safely modulate systemic and cardiac inflammation in patients with cardiac sarcoidosis. Furthermore, the current trial demonstrates an innovative collaborative approach to clinical trial development in a rare, understudied disease.

## Methods

### Design

**Fig. 1:**
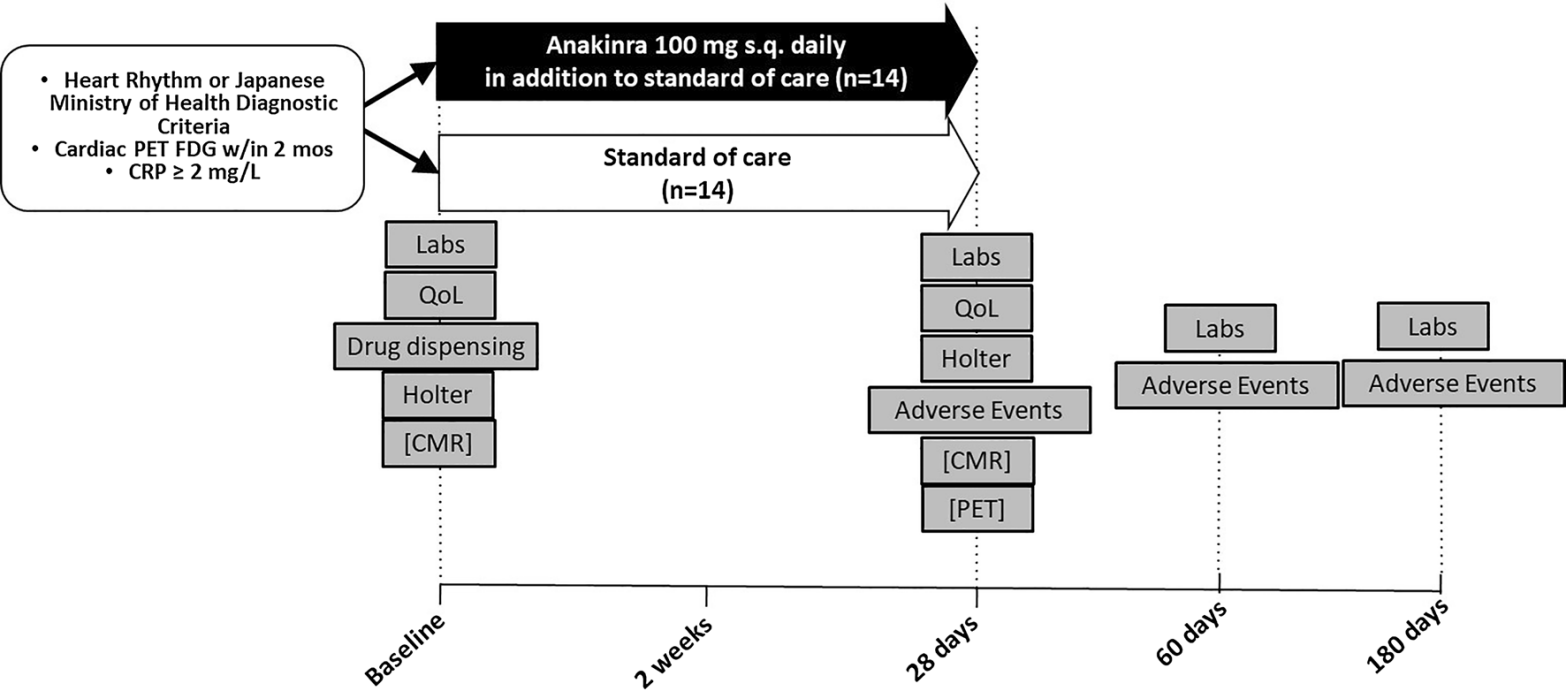
Design of the Multimodality Assessment of Granulomas in Cardiac Sarcoidosis—Anakinra Randomized Trial (MAGiC-ART). The overall study design is represented as a schematic. Abbreviations: CMR, cardiac magnetic resonance; CRP, C-reactive protein; HRS, Heart Rhythm Society; PET, positron emission tomography; QoL, quality of life. Procedures shown in brackets are additional testing done as part of the VCU Imaging Sub-study

The trial is a two-center, randomized, open-label clinical trial comparing administration of anakinra 100 mg daily on top of standard of care versus standard of care only for 28 days to determine whether IL-1 blockade with anakinra is associated with a biological signal or not, based on surrogate endpoints of changes in systemic inflammatory biomarkers (Fig. 1). We plan to enroll 28 subjects (age > 21 years) with a clinical diagnosis of cardiac sarcoidosis, cardiac uptake on FDG-PET within 2 months, C-reactive protein (CRP) high-sensitivity assay ≥ 2 mg/L at Virginia Commonwealth University (Richmond, VA) and University of Michigan (Ann Arbor, Michigan). Potential patients will be assessed for eligibility and sign an informed consent form prior to randomization and study drug administration. The following procedures will be performed during the screening: collection of medical history, recording of previous and concomitant therapy, collection of demographic data, lab work including CRP, baseline FDG-PET scan, and 24-h ECG Holter monitor. Patients who are part of the VCU Imaging sub-study will also undergo cardiovascular magnetic resonance imaging (CMR). The trial was registered prospectively with ClinicalTrials.gov on July 12, 2019, identifier NCT04017936. Institutional Review Board (IRB) approval was obtain at both centers using IRB reliance on the VCU IRB.

## Inclusion criteria

Patients with sarcoidosis will be screened according to the following criteria *(all 3 criteria must be met)* (Table 1):

1. Clinical diagnosis of cardiac sarcoidosis based on Heart Rhythm Society Diagnostic Criteria [16] or diagnostic guidelines for cardiac sarcoidosis based on New CS Guidelines in Japan or the Diagnostic guidelines for isolated cardiac sarcoidosis based on New CS Guidelines in Japan [17] [See inclusion criteria for details].
2. Cardiac fluoro-deoxyglucose uptake on recent PET (performed within the prior 60 days)
3. CRP high-sensitivity assay ≥ 2 mg/l (performed within the prior 60 days)

**Table 1:** Inclusion Criteria

Clinical diagnosis of cardiac sarcoidosis according to either the Heart Rhythm Society Diagnostic Criteria [16] based on one of the 2 diagnosis pathways (see below):
1. Histological diagnosis from myocardial tissue: cardiac sarcoidosis is diagnosed in the presence of non-caseating granuloma on histologic examination of myocardial tissue with no alternative cause identified (including negative stain for microorganisms—as applicable)
2. Clinical diagnosis from invasive and/or non-invasive studies: it is probable that there is cardiac sarcoidosis if there is (a) histological diagnosis of extracardiac sarcoidosis and (b) one or more of the following: steroid ± immunosuppressant responsive cardiomyopathy or heart block; unexplained reduction in LVEF (< 40%); unexplained sustained (spontaneous or induced) ventricular tachycardia; Mobitz type II 2nd degree or 3rd degree AV block; patchy uptake on dedicated cardiac PET (in a pattern consistent with cardiac sarcoidosis); late gadolinium enhancement on cardiac magnetic resonance (in a pattern consistent with cardiac sarcoidosis); positive gallium uptake (in a pattern consistent with cardiac sarcoidosis) and (c) other causes for the cardiac manifestation(s) have been reasonable excluded
or the diagnostic guidelines for cardiac sarcoidosis based on New CS Guidelines in Japan [17] (see below):
1. Histological diagnosis group (those with positive myocardial biopsy findings) Cardiac sarcoidosis is diagnosed histologically when endomyocardial biopsy or surgical specimens demonstrate non-caseating epithelioid granulomas
2. Clinical diagnosis group (those with negative myocardial biopsy findings or those not undergoing myocardial biopsy)
The patient is clinically diagnosed as having sarcoidosis:
(1) When epithelioid granulomas are found in organs other than the heart and clinical findings strongly suggestive of the above-mentioned cardiac involvement are present (Table 1A); or
R(2) When the patient shows clinical findings strongly suggestive of pulmonary or ophthalmic sarcoidosis; at least 2 of the 5 characteristic laboratory findings of a sarcoidosis (Table 1B); and clinical findings strongly suggest the above-mentioned cardiac involvement (Table 1A)

A. Clinical findings defining cardiac involvement
Cardiac findings should be assessed based on the major criteria and the minor criteria. Clinical findings that satisfy the following 1) or 2) strongly suggest the presence of cardiac involvement
1. 2 or more of the 5 major criteria (a)-(e) are satisfied
2. 1 of the 5 major criteria (a)-(e) and 2 or more of the 3 minor criteria (f)-(h) are satisfied
1. Major criteria:
a. High-grade AV block (including complete AV block) or fatal ventricular arrhythmia (e.g., sustained VT and VF)
b. Basal thinning of the ventricular septum or abnormal ventricular wall anatomy (ventricular aneurysm, thinning of the middle or upper ventricular septum, regional wall thickening
c. Left ventricular contractile dysfunction (LVEF < 50%)
d. ^67^Ga citrate scintigraphy or ^18^F-FDG PET reveals abnormally high tracer accumulation in the heart
e. Gadolinium-enhanced MRI revealed delayed contrast enhancement of the myocardium
2. Minor criteria
f. Abnormal ECG findings: Ventricular arrhythmias (nonsustained VT, multifocal or frequent premature ventricular contractions, bundle branch block, axis deviation, or abnormal Q waves
g. Perfusion defects on myocardial perfusion scintigraphy (SPECT)
h. Endomyocardial biopsy: Monocyte infiltration and moderate or severe myocardial interstitial fibrosis

B. Characteristic laboratory findings of sarcoidosis
1. Bilateral hilar lymphadenopathy
2. High serum angiotensin-converting (ACE) activity or elevated serum lysozyme levels
3. High serum soluble interleukin-2 receptor (sIL-2R) levels
4. Significant tracer accumulation in 67 Ga citrate scintigraphy or 18F-FDG PET
5. A high percentage of lymphocytes with a CD4CD8 ration of > 3.5 in BAL fluid

or the Diagnostic guidelines for isolated cardiac sarcoidosis based on New CS Guidelines in Japan [17]
Diagnostic guidelines for isolated cardiac sarcoidosis
*Prerequisites*
1. No clinical findings characteristic of sarcoidosis are observed in any organs other than the heart. (The patient should be examined in detail for respiratory, ophthalmic, and skin involvement of sarcoidosis. When the patient is symptomatic, other etiologies that can affect the corresponding organs must be ruled out.)
2. ^67^Ga scintigraphy or ^18^F-FDG PET reveals no abnormal tracer accumulation in any organs other than the heart
3. A chest CT scan reveals no shadow along the lymphatic tracts in the lungs or no hilar and mediastinal lymphadenopathy (minor axis > 10 mm)
*(1) Histological diagnosis group*
Isolated cardiac sarcoidosis is diagnosed histologically when endomyocardial biopsy or surgical specimens demonstrate non-caseating epithelioid granulomas
*(2) Clinical diagnosis group*
Isolated cardiac sarcoidosis is diagnosed clinically when criterion (d) and at least 3 other major criteria (a)-(e) are satisfied. (Table 1A)
2. Cardiac fluoro-deoxyglucose uptake on recent PET (performed within the prior 60 days)
3. CRP high-sensitivity assay ≥ 2 mg/l (performed within the prior 60 days)

Exclusion criteria are shown in Table 2. Patients may not be on acute or chronic immunosuppressive therapies *excluding* stable (> 1 month) oral corticosteroids at a dose of prednisone less than 0.5 mg/kg/day and/or methotrexate. Patients may not be on any biologic immunosuppressive agents.

**Table 2:** Exclusion Criteria

Subjects will not be eligible if they meet any one of the following exclusion criteria
1. Age < 21 years;
2. Pregnancy;
3. Inability to obtain consent from patient or legally authorized representative;
4. Contraindications to treatment with Anakinra (Kineret)(i.e. prior allergic reaction to the drug or to E. coli derived products or severe allergy to latex);
5. Severe anemia (*Hgb* < *8 g/dl—due to the need of more frequent blood sampling with this study*)
6. Acute or chronic active infections (*not including treated/cured HCV with negative viral load*)
7. Acute or chronic inflammatory disease or immunosuppressive therapies (*excluding stable [*> *1 month] oral corticosteroids at a dose of prednisone less than 0.5 mg/kg/day or methotrexate*)
8. Active acute or chronic psychiatric illness that in the opinion of the investigator may prevent from complying with study instructions;
9. Limited English Proficiency that in the opinion of the investigator may prevent from understanding the content of the informed consent form or safely completing the study procedures
10. Live vaccination within the prior month
11. Neutropenia (defined as absolute neutrophil count < 1,500/ml or < 1,000/ml if subject is African American)
12. History of malignancy within the prior 5 years (with exception of basal cell skin cancer, carcinoma in-situ of the cervix or low risk prostate cancer after curative therapy)
13. Participation in another concurrent intervention study within 30 day or treatment with an investigational drug within 5 half-lives prior to randomization
14. Severe kidney disease (GFR < 30 mL/min/1.73m^2^)
15. Evidence of COVID-19 within the last 60 days or recent (21 days) exposure to close personal contact with COVID-19
16. Chronic, moderate-to-severe kidney disease (GFR < 60 mL/min/1.73m^2^) or acute kidney injury, or history of severe hypersensitivity reactions to gadolinium-based contrast agents—for VCU Imaging Sub-study, patients may participate but will not undergo CMR

### Patient randomization and treatment allocation

Consented patients will be randomized in 1:1 to anakinra plus standard of care (SOC) vs. SOC only in an open label fashion (Fig. 1). Randomization will be handled by a member of the research team not involved in the consenting process using a dedicated randomization algorithm, stratified by center (VCU / University of Michigan) and by race/ethnicity (Caucasian / others). Assessment of ECG Holter and of imaging, for the sub-study, will be performed by operators who are blinded to treatment allocation.

After completion of all baseline testing, patients randomized to anakinra will be given a 28-day supply of anakinra and will also receive instruction from the investigators regarding self-injection technique. Adherence to the investigational treatment will be addressed by count of syringes and completion of all study visits. All concomitant medications will also be recorded at each clinic visit.

The patients are followed out through 180 days, but after 28 days, treatment may be changed at the discretion of the treating physicians. We will keep track of the medications at each visit. The primary endpoint is limited to 28 days and additional assessments are for safety purposes only.

### Outcomes

#### Efficacy assessments

The primary endpoints include the number of subjects enrolled, stratified by race/ethnicity [Feasibility Endpoint] and the interval changes in inflammatory biomarkers (hs-CRP and IL-6) after 4 weeks of treatment with anakinra [Efficacy Endpoints] (Table 3). The secondary endpoints include clinical outcomes, 24-h Holter monitor arrhythmia assessment, quality of life assessment using Sarcoidosis Assessment Tool [18], and for the imaging sub-study, change of tracer uptake intensity of FDG PET and change in LV ejection fraction and late gadolinium enhancement on CMR. The primary and secondary endpoints are shown in Table 3.

**Table 3:** The primary endpoints, including the feasibility endpoint and efficacy endpoints, and secondary endpoints for the MAGiC-ART trial are shown

Primary Endpoints	Feasibility Endpoint	Number of subjects enrolled, stratified by race/ethnicity
	Efficacy Endpoints	Interval changes in inflammatory biomarkers (CRP and IL-6) after 4 weeks of treatment with anakinra
Secondary Endpoints	Clinical Outcomes	Incidence of death (cardiac and non-cardiac) Hospitalization (for cardiac and non-cardiac reasons) Change in medication use for sarcoidosis (number and doses) Change in medication use for heart disease (number and doses) Adverse events at 28, 60, and 180 days
	24 Hour Holter	Number, duration, and rate of sustained VT Number, duration, and rate of non-sustained VT Absolute number and percentage of premature ventricular contractions Number and type of AV blocks Number and duration of sinus pauses Occurrence of atrial arrhythmias
	Quality of Life	Change in Sarcoidosis Assessment Tool scores
[Imaging Substudy @ VCU]	Cardiac Magnetic Resonance	Change in left ventricular ejection fraction Change in late gadolinium enhancement
	Cardiac FDG-PET	Change in intensity of tracer uptake

### Statistical analysis

#### Sample size determination

Using an informatics-based tool by TriNetX, a global health research network able to perform real-time analyses on electronic medical records of > 22 million patients from 19 health care organizations, we ran a retrospective analysis of patients seen in the Cardiac Sarcoidosis Clinic at VCU in the past year. We identified 30 subjects with cardiac sarcoidosis who had CRP 3–20 mg/L (we excluded patients with CRP > 20 mg/L as they may have had a concomitant bacterial infection, and therefore might be excluded upon screening). The mean age was 55 years (range 37–82), 55% were men, and 77% were African American. The CRP was 7.64 ± 3.74 mg/L (mean ± standard deviation). We predict a reduction of CRP by > 60% in the anakinra group and no change in standard of care only group. Table 4 shows estimated power for CRP reduction between 50 and 75% in the active treatment group (on top of standard of care changes, which are expected to be close to 0), and we evaluated 3 different standard deviation (SD) values starting with 3.74 mg/l which was obtained from our VCU cohort and exploring 0.9 × and 1.1 × SD. With assumption for a 60% reduction in the anakinra group, and a standard deviation of 3.74 mg/L, we predict a 90% power for a sample size of 28 subjects.

**Table 4:** Estimated power calculation for CRP reduction between 50 and 75% in the active treatment group is shown, evaluated with 3 different standard deviation (SD) values

Power analysis	CRP reduction (estimated mean 7.64 mg/l)					
N = 28	50%	55%	60%	65%	70%	75%
SD 3.37 mg/l	85%	91%	95%	97%	99%	> 99%
SD 3.74 mg/l	77%	84%	90%	94%	97%	98%
SD 4.11 mg/l	69%	77%	84%	89%	93%	96%

### Primary outcome analysis

We hypothesize that anakinra will reduce systemic inflammation as shown by CRP. CRP will be measured with a widely available high-sensitivity assay able to detect values in the normal range (< 2 mg/l). CRP will be measured at enrollment and again at 28 ± 3 days. We will measure the interval change of CRP within the anakinra treated group and also compare the change between groups using an ANOVA for repeated measures in which we assess for the significance of the interaction of treatment allocation and time interval (time x group interaction). Additional exploratory endpoints will include absolute CRP values at 28 days, and the percentage of patients with CRP ≤ 2 mg/L at each time point. The interval change in CRP levels from baseline to 28 days will be expressed as median and interquartile range.

### Secondary outcome analysis

We will measure changes in IL-6, an additional inflammatory biomarker as a secondary endpoint. Changes in Sarcoidosis Assessment Tool QoL scores will also be considered a secondary endpoint. Additional secondary endpoints to be completed as part of the imaging sub-study at VCU include assessment of myocardial inflammation with cardiac FDG-PET and myocardial edema with T2 mapping on CMR.

### Safety analysis

Safety labs, including complete blood count with differential, comprehensive metabolic panel, and urine pregnancy at randomization as applicable, will be performed at baseline, day 28, and day 60. A Data and Safety Monitoring Board (DSBM) was formed to monitor the progress of the present trial. The DSMB is composed by experts in cardiology, electrophysiology, pulmonary disease, immunology, and clinical trial design and methodology. The DSMB will meet regularly to review safety data every 6 months or sooner in case of a serious unexpected adverse event that is considered to be possibly, probably, or definitely related to the research. All meetings, and specifically any action taken by the committee and the reasons for the actions, will be recorded. These documents will include any data summaries or analyses provided to the DSMB and will remain confidential until the study is concluded. No interim efficacy analyses are planned.

Interim safety analyses will be performed at each meeting. The DSMB will be provided with data related to adverse events. The DSMB may choose to stop enrollment on the basis of safety data observed. If safety concerns are found, further enrollment will not be allowed until issues are resolved. If no safety concerns are found, enrollment will continue until the target sample size is reached. The following pre-specified halting rules are provided to the DSMB as guidance. The decision regarding continuation or termination of the study will be solely based on safety data, and will be made by the DSMB. Interim analyses will be performed by the DSMB. The study will be temporarily suspended if any of the following conditions are met:

- *Any serious adverse event that probably or definitely related to the investigational product*;
- The number of subjects experiencing a *serious* adverse event considered *unrelated or only possibly related* to the investigational product exceeds 50% (after the first 3 or more subjects are enrolled);
- The number of subjects experiencing *either a serious adverse event considered unrelated or only possibly related* to the investigational product or *a non-serious adverse event of at least moderate intensity that is probably or definitely related* to the investigational product exceeds 75%.

If any of these conditions are met, the enrollment is halted until the DSMB is given the opportunity to review the data. Once assessed by the DSMB, a discussion with the PI and the Sponsor will be held prior to the final decision to resume or permanently stop the study.

### Progress to date

The study began enrollment in February 2020. Enrollment was paused from March 2020 through August 2020 due to the COVID-19 pandemic. A total of 14 patients have been enrolled and 12 randomized from two centers, VCU and University of Michigan. Of the 12 subjects randomized, 9 (75%) were female and 3 (25%) male, and 6 (50%) White and 6 (50%) Black.

## Discussion

Cardiac sarcoidosis is a potentially life-threatening disease that can cause ventricular arrhythmias, conduction system block and heart failure and as of yet has limited therapeutic options. Corticosteroids are the standard treatment for sarcoidosis but the evidence for efficacy from clinical trials is largely lacking and their use is hindered by significant toxicities.

Methotrexate is the most commonly used steroid-sparing agent, but takes up to three months to reach maximal clinical effect. Side effects are also common and include bone marrow suppression, liver toxicity, and gastrointestinal intolerance. While effective in controlling rheumatologic symptoms, methotrexate is ineffective in preventing cardiovascular outcomes in patients with heart disease—as shown by the neutral results of the large *Cardiovascular Inflammation Reduction Trial* (*CIRT*) [19]. There have been no clinical trials of methotrexate in patients with cardiac sarcoidosis—*the use of methotrexate in cardiac sarcoidosis is therefore also empirical*.

Tumor necrosis factor alpha (TNF-α) inhibitors, such as the monoclonal antibody infliximab, have also been used to treat refractory sarcoidosis [20], including some cases of cardiac sarcoidosis, however, infliximab use in patients with heart failure has been associated with a dose-dependent increased risk of heart failure-related death [21]. Infliximab is also associated with an increased risk of hematologic cancer, and a > twofold increased risk of opportunistic infection [22, 23]. Infliximab and other TNF-α inhibitors are currently reserved for refractory cases.

IL-1 blockade has been evaluated as a potential therapeutic target in acute myocardial infarction and heart failure [15]. Anakinra, recombinant human IL-1 receptor antagonist, that blocks IL-1α and IL-1β, has been FDA approved for the treatment of rheumatoid arthritis since 2001. In a series of phase II studies, treatment with anakinra was shown to be safe and associated with reduced incidence of heart failure in patients with ST-segment elevation MI (STEMI) [24], and with improved cardiorespiratory fitness and quality of life in patients with systolic heart failure [25, 26]. In the large CANTOS trial of 10,061 patients with previous myocardial infarction and elevated CRP (> 2 mg/L), canakinumab, a monoclonal antibody blocking interleukin-1β, led to a 15% significantly lower rate of atherothrombotic events [27], a 36% reduction in need for coronary revascularization [27], and a dose-dependent reduction in hospitalization for heart failure [28]. In all instances, the clinical improvement correlated with reduction of the systemic inflammatory biomarkers, namely CRP, showing a dose–response relationship between CRP reduction and benefits observed.

While IL-1 plays a role in the pathogenesis of granuloma formation in sarcoidosis, IL-1 blockade has never been evaluated as a potential therapeutic agent for cardiac sarcoidosis. IL-1 blockade represents a viable therapeutic strategy in heart disease. The beneficial cardiovascular effects seen with IL-1 blockers (anakinra and canakinumab) are in stark contrast with the lack of benefits with methotrexate, and the dose-dependent increase in heart failure-related mortality seen with the TNF-α blocker (infliximab). Inflammatory biomarkers, such as CRP, are used as surrogates for IL-1 activity and have been shown to predict adverse outcomes in patients across a wide spectrum of cardiovascular disease. In this pilot study, CRP and IL-6 were chosen as endpoints because a decrease in biomarkers, while not sufficient to show clinical benefit, will indicate potential benefit and support future trials to investigate further.

### Safety profile of IL-1 blockade

IL-1 blockers have been widely studied in patients with heart disease, showing a low rate of predictable complications, free of off-target effects, making them an ideal anti-inflammatory agent to be studied in CS. The CANTOS trial is the largest clinical trial of any cytokine blocker or biologic performed to date with more than 20,000-patient-year exposure, providing the opportunity to determine with precision the magnitude of increased risk of infection expected with IL-1 blockade. An excess of 1 fatal infection was seen for every 1000 patient-year exposure with canakinumab. Anakinra, being an inhibitor with shorter half-life (4.5 h versus 25 days for canakinumab), is associated with a much lower rate of serious or fatal infections. The infectious risk with IL-1 blockers is several-fold lower than with other immune-modulating drugs, like infliximab. Also, in contrast to infliximab (TNF-α blocker), there were no excess opportunistic infections (1.5 versus 1.8 per 1000 person-year for canakinumab and placebo, respectively). More recently IL-1 blockers have been studied in patients with COVID-19 pneumonia showing an acceptable safety profile [29]. Not surprisingly, canakinumab was also associated with a significant reduction in the incidence or worsening of arthritis in the CANTOS trial that may prove of particular benefit in patients with sarcoidosis. IL-1 blockers, like anakinra and canakinumab, are also well-tolerated across a wide spectrum of glomerular filtration rates, require minimal dose adjustment, and do not negatively affect renal flow, filtration, or tubular function. [30–32].

### Imaging in cardiac sarcoidosis

The lack of efficacious treatment for CS may also be hindered by the challenges of detecting and monitoring cardiac-specific involvement in sarcoidosis. PET imaging is often used, and perfusion defects and FDG uptake on PET can identify patients at higher risk of death or VT [33]. PET has the advantage that it can be used in patients with impaired renal function and can show disease activity that can guide the need for and response to immunosuppressive therapy, but its use is limited by spatial resolution and risk of repeated exposure to ionizing radiation [34]. CMR imaging is a non-invasive imaging methodology that does not expose patients to ionizing radiation and is less expensive than PET imaging [34]. Inflammation may be identified and quantified with CMR using late gadolinium enhancement (LGE) imaging or T2 mapping [35]. The most common CMR pattern in CS is multi-focal and patchy LGE with sparing of the endocardial border [36–38]. Importantly, LGE is associated with increased risk of all-cause mortality and arrhythmogenic events in patients with CS [39]. In the Imaging sub-study, we will evaluate newer CMR techniques, including native T1 mapping, T2 mapping and extracellular volume mapping, which allow deeper interrogation into myocardial tissue changes.

### Challenges of research in cardiac sarcoidosis

To date, there have been no published randomized controlled clinical trials in cardiac sarcoidosis. Sarcoidosis is a relatively rare, understudied disease with heterogenous manifestations. In the current study, we build on the resources available at the two CTSA hubs at VCU and University of Michigan as well as a network of research collaborators established in the Cardiac Sarcoidosis Consortium to demonstrate the potential for recruiting and retaining CS patients.

The current study has also faced the challenge of maximizing patient safety during the worldwide COVID-19 pandemic. Patient enrollment was halted from March to August 2020 to decrease risk to potentially immunocompromised CS patients from attending study visits in the hospital and risking potential virus exposure. The original study design was one of double-blind placebo controlled utilizing placebo syringes to be provided by the manufacturer of Anakinra (Kineret®) and that were indistinguishable from the anakinra syringes. Due to an unexpected shortage of syringes for placebo during the COVID-19 pandemic [40], the use of placebo syringes, initially to be supplied by the manufacturer of Kineret®, became unavailable for this trial. The trial design was thus changed to a randomized open-label anakinra plus SOC versus SOC alone. CRP and other laboratory tests are largely placebo insensitive and interpretation of data from ECG Holters, FDG-PET, and CMR will continue to be performed by operators blinded to treatment allocation in a prospective randomized open blinded end-point (PROBE) design fashion.

### Disparities in sarcoidosis

CS, and sarcoidosis in general, affects female and Blacks disproportionately [1] yet these groups are historically underrepresented in cardiovascular clinical trials [41], creating large gaps in knowledge. Despite increasing awareness of the underrepresentation of these groups, women and minorities, particularly blacks, have continued to be inadequately represented in pivotal cardiometabolic clinical trials over the past decade [41]. Awareness and sensitization campaigns have been performed to help fill this gap, and studies specifically designed to understand and target challenges in recruitment and enrollment of unrepresented populations in clinical trials is of the utmost interest, especially in the context of diseases where large knowledge gaps already exist.

### Limitations

There are several important limitations to note about the design of this trial. Due to the chronic nature of cardiac sarcoidosis, patients typically require long-term treatment. However, we chose a 28-day time period to demonstrate feasibility and to look for safety and a biological signal that will serve as the basis for future studies that will be longer in duration and include more patients. Clinical trials in CS are lacking and we chose to pursue an initial small scale pilot study as a first essential step. The study was changed from a blinded study design to an open-label design due to the lack of availability of placebo syringes indistinguishable from anakinra due to clinical trials of anakinra during the ongoing COVID-19 pandemic. While biomarker data are largely placebo insensitive, the quality of life scores may be altered by patient perceptions and expectations from the trial drug and the data will be interpreted with appropriate caution. However, since no randomized clinical trials have been completed in patients with CS, we feel the QoL data will be helpful to further characterize our study patients and identify barriers to recruitment and retention. CS patients often have multi-organ system disease and face multiple physical, emotional and social challenges, all of which may be exacerbated during a worldwide pandemic. While the open-label trial design limits our comparison of the two patient groups, we feel that the QoL data is nonetheless extremely valuable to designing and optimizing the success of future trials. The imaging substudy is being performed only at one center and will therefore yield a small amount of data. The imaging data thus will be considered as hypothesis-generating.

## Conclusion

The ongoing pilot MAGIC-ART study will ascertain the feasibility of recruitment and enrollment of patients with cardiac sarcoidosis in two CTSA hubs and WASOG centers and test the tolerability of treatment with anakinra to assess how IL-1 blockade modulates systemic inflammation.

## Data Availability

A simplified and fully de-identified database will be made available for sharing in accordance with requirements for NHLBI data repository datasets and associated documentation for submission to the Biological Specimen and Data Repository Information Coordinating Center (BioLINCC) and the NHLBI Policy for Data Sharing from Clinical Trials and Epidemiological Studies, and in accordance with the Guidelines for NHLBI Data Set Preparation, within 3 years of completion of the study.

## Abbreviations

CMR: Cardiac magnetic resonance
COVID-19: Coronavirus disease 2019
CRP: C-reactive protein
CS: Cardiac sarcoidosis
CSC: Cardiac Sarcoidosis Consortium
CTSA: Clinical and Translational Science Award
DSMB: Data and Safety Monitoring Board
FDA: Food and Drug Administration
FDG: Fluoro-deoxyglucose
IL-1: Interleukin-1
IL-6: Interleukin-6
IL-18: Interleukin-18
IRB: Institutional Review Board
LGE: Late gadolinium enhancement
LV: Left ventricle
NF-kB: Nuclear transcription factor-kB
PET: Positron emission tomography
SOC: Standard of care
STEMI: ST-segment elevation myocardial infaction IL-1, interleukin-1
TNF-α: Tumor necrosis factor-α
VCU: Virginia Commonwealth University
WASOG: World Association for Sarcoidosis and Other Granulomatous Disorders

## References

[1] Rybicki BA, Major M, Popovich J, Maliarik MJ, Iannuzzi MC (1997). Racial differences in sarcoidosis incidence: a 5-year study in a health maintenance organization. Am J Epidemiol.

[2] Cozier YC, Berman JS, Palmer JR, Boggs DA, Serlin DM, Rosenberg L (2011). Sarcoidosis in black women in the United States: data from the Black Women's Health Study. Chest.

[3] Valeyre D, Prasse A, Nunes H, Uzunhan Y, Brillet PY, Müller-Quernheim J (2014). Sarcoidosis. Lancet (London, England).

[4] Grutters JC, van den Bosch JM (2006). Corticosteroid treatment in sarcoidosis. Eur Respir J.

[5] Israel HL, Gottlieb JE (1995). Outcome of the treatment for sarcoidosis. Am J Respir Crit Care Med.

[6] Izumi T (1988). Sarcoidosis in Kyoto (1963–1986). Sarcoidosis.

[7] Paramothayan NS, Lasserson TJ, Jones PW (2005). Corticosteroids for pulmonary sarcoidosis. Cochrane Database Syst Rev.

[8] Dinarello CA (2011). Interleukin-1 in the pathogenesis and treatment of inflammatory diseases. Blood.

[9] Toldo S, Abbate A (2018). The NLRP3 inflammasome in acute myocardial infarction. Nat Rev Cardiol.

[10] Toldo S, Mezzaroma E, Mauro AG, Salloum F, Van Tassell BW, Abbate A (2015). The inflammasome in myocardial injury and cardiac remodeling. Antioxid Redox Signal.

[11] Mikuniya T, Nagai S, Takeuchi M, Mio T, Hoshino Y, Miki H, Shigematsu M, Hamada K, Izumi T (2000). Significance of the interleukin-1 receptor antagonist/interleukin-1 beta ratio as a prognostic factor in patients with pulmonary sarcoidosis. Respiration.

[12] Kasahara K, Kobayashi K, Shikama Y, Yoneya I, Soezima K, Ide H, Takahashi T (1988). Direct evidence for granuloma-inducing activity of interleukin-1. Induction of experimental pulmonary granuloma formation in mice by interleukin-1-coupled beads. Am J Pathol.

[13] Kron J, Mauro AG, Bonaventura A, Toldo S, Salloum FN, Ellenbogen KA, Abbate A (2019). Inflammasome formation in granulomas in cardiac sarcoidosis. Circul Arrhythmia Electrophysiol.

[14] Libby P (2017). Interleukin-1 beta as a target for atherosclerosis therapy: Biological basis of CANTOS and beyond. J Am Coll Cardiol.

[15] Buckley LF, Abbate A (2018). Interleukin-1 blockade in cardiovascular diseases: a clinical update. Eur Heart J.

[16] Birnie DH, Sauer WH, Bogun F, Cooper JM, Culver DA, Duvernoy CS, Judson MA, Kron J, Mehta D, Cosedis Nielsen J, Patel AR, Ohe T, Raatikainen P, Soejima K (2014). HRS expert consensus statement on the diagnosis and management of arrhythmias associated with cardiac sarcoidosis. Heart Rhythm.

[17] Terasaki FYK (2017). New guidelines for diagnosis of cardiac sarcoidosis in Japan. An Nucl Cardiol.

[18] Judson MA, Mack M, Beaumont JL, Watt R, Barnathan ES, Victorson DE (2015). Validation and important differences for the Sarcoidosis Assessment Tool. A new patient-reported outcome measure. Am J Respir Crit Care Med.

[19] Ridker PM, Everett BM, Pradhan A, MacFadyen JG, Solomon DH, Zaharris E, Mam V, Hasan A, Rosenberg Y, Iturriaga E, Gupta M, Tsigoulis M, Verma S, Clearfield M, Libby P, Goldhaber SZ, Seagle R, Ofori C, Saklayen M, Butman S, Singh N, Le May M, Bertrand O, Johnston J, Paynter NP, Glynn RJ (2018). Low-Dose methotrexate for the prevention of atherosclerotic events. N Engl J Med.

[20] Gilotra NA, Wand AL, Pillarisetty A, Devraj M, Pavlovic N, Ahmed S, Saad E, Solnes L, Garcia C, Okada DR, Constantinescu F, Mohammed SF, Griffin JM, Kasper EK, Chen ES, Sheikh FH (2021). Clinical and imaging response to tumor necrosis factor alpha inhibitors in treatment of cardiac sarcoidosis: a multicenter experience. J Cardiac Fail.

[21] Chung ES, Packer M, Lo KH, Fasanmade AA, Willerson JT (2003). Randomized, double-blind, placebo-controlled, pilot trial of infliximab, a chimeric monoclonal antibody to tumor necrosis factor-alpha, in patients with moderate-to-severe heart failure: results of the anti-TNF Therapy Against Congestive Heart Failure (ATTACH) trial. Circulation.

[22] Bongartz T, Sutton AJ, Sweeting MJ, Buchan I, Matteson EL, Montori V (2006). Anti-TNF antibody therapy in rheumatoid arthritis and the risk of serious infections and malignancies: systematic review and meta-analysis of rare harmful effects in randomized controlled trials. JAMA.

[23] Caporali R, Crepaldi G, Codullo V, Benaglio F, Monti S, Todoerti M, Montecucco C (2018). 20 years of experience with tumour necrosis factor inhibitors: what have we learned?. Rheumatology (Oxford).

[24] Abbate A, Kontos MC, Abouzaki NA, Melchior RD, Thomas C, Van Tassell BW, Oddi C, Carbone S, Trankle CR, Roberts CS, Mueller GH, Gambill ML, Christopher S, Markley R, Vetrovec GW, Dinarello CA, Biondi-Zoccai G (2015). Comparative safety of interleukin-1 blockade with anakinra in patients with ST-segment elevation acute myocardial infarction (from the VCU-ART and VCU-ART2 pilot studies). Am J Cardiol.

[25] Van Tassell BW, Arena RA, Toldo S, Mezzaroma E, Azam T, Seropian IM, Shah K, Canada J, Voelkel NF, Dinarello CA, Abbate A (2012). Enhanced interleukin-1 activity contributes to exercise intolerance in patients with systolic heart failure. PLoS ONE.

[26] Van Tassell BW, Canada J, Carbone S, Trankle C, Buckley L, Oddi Erdle C, Abouzaki NA, Dixon D, Kadariya D, Christopher S, Schatz A, Regan J, Viscusi M, Del Buono M, Melchior R, Mankad P, Lu J, Sculthorpe R, Biondi-Zoccai G, Lesnefsky E, Arena R, Abbate A (2017). Interleukin-1 blockade in recently decompensated systolic heart failure: results from REDHART (Recently Decompensated Heart Failure Anakinra Response Trial). Circ Heart Fail.

[27] Ridker PM, Everett BM, Thuren T, MacFadyen JG, Chang WH, Ballantyne C, Fonseca F, Nicolau J, Koenig W, Anker SD, Kastelein JJP, Cornel JH, Pais P, Pella D, Genest J, Cifkova R, Lorenzatti A, Forster T, Kobalava Z, Vida-Simiti L, Flather M, Shimokawa H, Ogawa H, Dellborg M, Rossi PRF, Troquay RPT, Libby P, Glynn RJ, Group CT (2017). Antiinflammatory therapy with canakinumab for atherosclerotic disease. N Engl J Med.

[28] Everett BM, Cornel J, Lainscak M, Anker SD, Abbate A, Thuren T, Libby P, Glynn RJ, Ridker PM (2018). Anti-inflammatory therapy with canakinumab for the prevention of hospitalization for heart failure. Circulation.

[29] Sedhai YR, Sears M, Vecchiè A, Bonaventura A, Greer J, Spence K, Tackett H, Turner J, Pak M, Patel N, Black M, Wohlford G, Clary RE, Duke C, Hardin M, Kemp H, Priday A, Sims EK, Mihalick V, Ho AC, Ibe I, Harmon M, Markley R, Van Tassell B, Abbate A (2021). Clinical trial enrollment at a rural satellite hospital during COVID-19 pandemic. J Clin Transl Sci.

[30] Ridker PM, MacFadyen JG, Everett BM, Libby P, Thuren T, Glynn RJ (2018). Relationship of C-reactive protein reduction to cardiovascular event reduction following treatment with canakinumab: a secondary analysis from the CANTOS randomised controlled trial. Lancet (London, England).

[31] Van Tassell BW, Arena R, Biondi-Zoccai G, Canada JM, Oddi C, Abouzaki NA, Jahangiri A, Falcao RA, Kontos MC, Shah KB, Voelkel NF, Dinarello CA, Abbate A (2014). Effects of interleukin-1 blockade with anakinra on aerobic exercise capacity in patients with heart failure and preserved ejection fraction (from the D-HART pilot study). Am J Cardiol.

[32] Buckley LF, Canada JM, Carbone S, Trankle CR, Kadariya D, Billingsley H, Wohlford GF, Kirkman DL, Abbate A, Van Tassell BW (2019). Potential role for interleukin-1 in the cardio-renal syndrome. Eur J Heart Fail.

[33] Blankstein R, Osborne M, Naya M, Waller A, Kim CK, Murthy VL, Kazemian P, Kwong RY, Tokuda M, Skali H, Padera R, Hainer J, Stevenson WG, Dorbala S, Di Carli MF (2014). Cardiac positron emission tomography enhances prognostic assessments of patients with suspected cardiac sarcoidosis. J Am Coll Cardiol.

[34] Schatka I, Bengel FM (2014). Advanced imaging of cardiac sarcoidosis. J Nucl Med.

[35] Thavendiranathan P, Walls M, Giri S, Verhaert D, Rajagopalan S, Moore S, Simonetti OP, Raman SV (2012). Improved detection of myocardial involvement in acute inflammatory cardiomyopathies using T2 mapping. Circ Cardiovasc Imaging.

[36] Smedema JP, Snoep G, van Kroonenburgh MP, van Geuns RJ, Dassen WR, Gorgels AP, Crijns HJ (2005). Evaluation of the accuracy of gadolinium-enhanced cardiovascular magnetic resonance in the diagnosis of cardiac sarcoidosis. J Am Coll Cardiol.

[37] Patel MR, Cawley PJ, Heitner JF, Klem I, Parker MA, Jaroudi WA, Meine TJ, White JB, Elliott MD, Kim HW, Judd RM, Kim RJ (2009). Detection of myocardial damage in patients with sarcoidosis. Circulation.

[38] Cummings KW, Bhalla S, Javidan-Nejad C, Bierhals AJ, Gutierrez FR, Woodard PK (2009). A pattern-based approach to assessment of delayed enhancement in nonischemic cardiomyopathy at MR imaging. Radiographics.

[39] Coleman GC, Shaw PW, Balfour PC, Gonzalez JA, Kramer CM, Patel AR, Salerno M (2017). Prognostic value of myocardial scarring on CMR in patients with cardiac sarcoidosis. JACC Cardiovasc Imaging.

[40] Moeller WJ, Potere N, Bonaventura A, Vecchiè A, Sedhai YR, Caricchio R, Abbate A (2021). Use of placebo in clinical trials in COVID-19 pandemic times: considerations on pros, cons, challenges and limitations. Minerva Med.

[41] Khan MS, Shahid I, Siddiqi TJ, Khan SU, Warraich HJ, Greene SJ, Butler J, Michos ED (2020). Ten-year trends in enrollment of women and minorities in pivotal trials supporting recent US Food and Drug Administration Approval of Novel Cardiometabolic Drugs. J Am Heart Assoc.

